# Transcriptional Regulation of Chemokine Expression in Ovarian Cancer

**DOI:** 10.3390/biom5010223

**Published:** 2015-03-17

**Authors:** Bipradeb Singha, Himavanth R. Gatla, Ivana Vancurova

**Affiliations:** Department of Biological Sciences, St. John’s University, 8000 Utopia Parkway, New York, NY 11439, USA; E-Mails: bipradeb.singha10@my.stjohns.edu (B.S.); himavanthreddy.gatla12@my.stjohns.edu (H.R.G.)

**Keywords:** chemokines, interleukin-8, NFκB, ovarian cancer, transcriptional regulation

## Abstract

The increased expression of pro-inflammatory and pro-angiogenic chemokines contributes to ovarian cancer progression through the induction of tumor cell proliferation, survival, angiogenesis, and metastasis. The substantial potential of these chemokines to facilitate the progression and metastasis of ovarian cancer underscores the need for their stringent transcriptional regulation. In this Review, we highlight the key mechanisms that regulate the transcription of pro-inflammatory chemokines in ovarian cancer cells, and that have important roles in controlling ovarian cancer progression. We further discuss the potential mechanisms underlying the increased chemokine expression in drug resistance, along with our perspective for future studies.

## 1. Introduction

Chemokines are a family of cytokines that induce chemotaxis of target cells. Though they were originally discovered for their ability to induce leukocyte migration into the infected or injured sites, more recently, it became clear that they could also promote cancer progression [1,2,3,4,5,6,7,8,9]. In addition to inducing tumor cell proliferation, angiogenesis and metastasis, chemokines and their receptors regulate tumor cell differentiation and survival. Currently, the human chemokine network includes more than 45 known chemokines and 20 chemokine receptors. Based on the number and spacing of conserved N-terminal cysteine residues that form disulfide bonds, chemokines are divided into four groups: (X)C, CC, CXC, and CX3C [10,11,12].

Epithelial ovarian cancer (EOC) is among the leading causes of cancer death in women. Since most ovarian cancers relapse and become drug-resistant, the survival rates remain low. Progression of ovarian cancer (OC) has been associated with the increased expression and release of pro-inflammatory chemokines, which contribute to ovarian cancer development through their induction of tumor cell proliferation, survival, migration, and angiogenesis [13,14,15]. The chemokine expression by ovarian cancer cells is controlled at several levels that include transcriptional regulation, post-transcriptional regulation and regulation of mRNA stability, translation, and mechanisms regulating the cytokine intracellular storage, transport, and release. Table 1 summarizes chemokines produced by ovarian cancer cells. Several excellent reviews have addressed the physiological and cellular functions of these chemokines in ovarian cancer [9,16,17]. Thus, in this review, we focus instead on the main mechanisms that regulate transcription of these chemokines in ovarian cancer cells.

**Table 1:** Chemokines released by ovarian cancer cells.

Systematic Name	Alternate Human Names	Tissue/Cells	Reference
**CCL2**	Monocyte chemotactic protein 1 (MCP-1)	Tumor biopsies, serum and ascites	Negus *et al.*, 1995 [18] Milliken *et al.*, 2002 [19]
**CCL5**	RANTES	Tumor ascites, plasma and peritoneal fluid	Milliken *et al.*, 2002 [19] Negus *et al.*, 1997 [20]
**CCL11**	Eotaxin	Primary ovarian cancer cells obtained from ascites	Levina *et al.*, 2009 [21] Nolen *et al.*, 2010 [22]
**CCL25**	Thymus expressed chemokine (TECK)	Tumor tissue	Singh *et al.*, 2011 [23]
**CCL28**	Mucosae-associated epithelial chemokine (MEC)	Tumor tissue	Facciabene *et al*., 2011 [24]
**CXCL1**	Growth-regulated protein α (GRO-α)	Plasma and tumor ascites	Lee *et al.*, 2006 [25] Yang *et al.*, 2006 [26]
**CXCL2**	Growth-regulated protein β (GRO-β)	Ovarian cancer cell lines	Son *et al.*, 2007 [27] Kavandi *et al.*, 2012 [28]
**CXCL8**	Interleukin 8 (IL-8)	Tumor tissue, ascites, serum and cyst fluid	Lee *et al.*, 1996 [29] Xu *et al.*, 1999 [30]
**CXCL12**	Stromal cell-derived factor (SDF-1)	Tumor biopsies, tissues and ascites	Zou *et al.*, 2001 [31] Scotton *et al.*, 2002 [32]
**CXCL16**	Transmembrane chemokine CXCL16	Epithelial ovarian carcinoma tissue	Guo *et al.*, 2011 [33] Gooden *et al.*, 2014 [34]
**CX3CL1**	Fractalkine	Epithelial ovarian carcinoma tissue	Gaudin *et al.*, 2011 [35]
**XCL1/2**	Lymphotactin	Tumor ascites and ovarian cancer cell lines	Kim *et al.*, 2012 [36]

## 2. Mechanisms Regulating Chemokine Transcription in Ovarian Cancer Cells

### 2.1. Chemokine Regulation by NFκB and Epigenetic Acetylation

Chemokines are regulated at the transcriptional level by binding of transcription factors and repressors to gene promoter and enhancer regions. The transcription factors that control the expression of most inflammatory chemokines include the nuclear factor-κB (NFκB), activator protein-1 (AP-1) and the signal transducers and activators of transcription (STAT) family. The NFκB activity is constitutively increased in aggressive ovarian cancers, and inhibition of NFκB signaling suppresses angiogenesis and tumorigenicity of ovarian cancer cells and increases their sensitivity to chemotherapy and apoptosis [37,38,39,40]. The underlying mechanisms likely involve the NFκB-regulated chemokine expression, since several studies have demonstrated that the expression of CCL2, CXCL1, CXCL2, and IL-8/CXCL8 is mediated by NFκB in ovarian cancer cells [28,29,30,41].

The increased activity of NFκB in ovarian cancer cells is mediated by enzymes of the IκB kinase (IKK) complex, which phosphorylate the NFκB inhibitory protein, IκBα, resulting in IκBα proteasomal degradation and nuclear translocation of NFκB subunits [42,43,44,45]. In addition to phosphorylating IκBα, IKKs can also phosphorylate the NFκB subunits, particularly p65 [46]. While the cytoplasmic degradation of IκBα, resulting in the nuclear translocation of NFκB subunits, represents a general step in NFκB activation, the specificity of NFκB-regulated responses is mediated by the subunit composition of NFκB complexes and their post-translational modifications [47,48].

In addition to transcription factor binding to promoter sequences, chemokine expression is regulated by epigenetic modifications that include histone modifications as well as post-translational modifications of transcription factors, particularly the p65 subunit of NFκB. It is believed that while histone acetylation and acetylation of transcription factors induced by histone acetyl transferases (HATs) generally promotes transcriptional activation, hypoacetylation induced by histone deacetylase (HDAC) activity is associated with transcriptional repression. Since hypoacetylation of tumor suppressor genes by HDACs has been linked to tumor development, HDACs inhibitors are now being evaluated for their therapeutic effects in cancer, including ovarian cancer [49,50,51]. Clinical studies using HDAC inhibitors in the treatment of ovarian cancer are summarized in the recent elegant review by Khabele [52]. Numerous studies have shown that HDACs regulate chemokine expression in different cell types [53,54,55,56,57,58]; however, their role in the regulation of chemokine expression in ovarian cancer has yet to be documented.

### 2.2. Chemokine Modulation by Hypoxia and Metabolism

Ovarian cancer tissues and ascites are characterized by decreased oxygen content, which stabilizes the α-subunit of the transcription factor hypoxia-inducible factor-1 (Hif-1) [59]. Hif-1 responds to hypoxia by increasing the transcription of genes that promote survival in low-oxygen conditions, thus promoting angiogenesis and oncogenesis. Indeed, the increased expression of Hif-1 has been detected in epithelial ovarian cancer, and correlates with poor prognosis [60,61,62]. Hypoxia induces IL-8 [30], CXCL12 [63], and CCL28 [24] expression in ovarian cancer cells. The seminal study by Xu *et al*. [30] demonstrated that hypoxic conditions increase the IL-8 expression in ovarian cancer cells by increasing NFκB and AP-1 binding to IL-8 promoter. The mechanisms of how hypoxia increases the NFκB-dependent IL-8 transcription involve activation of the transforming growth factor beta-activated kinase 1 (TAK1), resulting in increased IKK activation, and p65 NFκB recruitment to the IL-8 promoter [64,65]. In addition, hypoxia induces a direct binding of Hif-1α to the hypoxia-response element (HRE) located next to the NFκB binding site in human IL-8 promoter, resulting in the increased IL-8 expression [66].

One of the consequences of Hif-1 activation is the increased expression of glycolytic genes, resulting in increased aerobic glycolysis, glucose consumption, and lactic acid production (Warburg effect) [67,68,69]. The high rate of glucose consumption and lactic acid production contributes to the acidification of the tumor environment and cancer progression. Xu *et al*. showed that acidic pH increases the IL-8 transcription by enhancing the binding of AP-1 and NFκB to IL-8 promoter in ovarian cancer cells [70]. In addition, in endothelial cells, lactate was shown to activate the NFκB-dependent IL-8 transcription by inducing degradation of IκBα [71]. The role of lactate and other metabolites of the glycolytic pathway in the regulation of pro-angiogenic chemokine expression in ovarian cancer cells is yet to be investigated, especially since recent studies have indicated high levels of aerobic glycolysis and lactate production in ovarian tumors [72,73].

While hyperglycemia and obesity are thought to be contributing factors to cancer development and progression, caloric restriction has been associated with reduced cancer incidence [74,75,76,77]. During reduced calorie intake or exercise, the body switches to obtaining energy from fatty acid oxidation, which results in ketone bodies production. Intriguingly, the recent study by Shimazu *et al*. [78] has demonstrated that the ketone body β-hydroxybutyrate (βOHB) is an endogenous and specific inhibitor of HDACs, and that administration of exogenous βOHB increases histone acetylation, correlating with changes in transcription. Since HDACs regulate chemokine transcription by both deacetylating histones and p65 NFκB [53,54,55,56,57,58], it will be important to analyze whether βOHB and other HDAC inhibitors regulate chemokine expression in ovarian cancer cells, and whether this is modulated by the metabolic state.

### 2.3. Chemokine Modulation by Chemotherapeutic Interventions

There is growing evidence that the increased chemokine expression by tumor cells modulates not only cancer development but also cancer responsiveness and resistance to chemotherapy [79]. A major contributor to the acquired chemoresistance of ovarian cancer cells is the increased expression of NFκB-dependent chemokines that is induced by the platinum-based drugs carboplatin and cisplatin, and by the mitotic inhibitors docetaxel and paclitaxel [29,80,81,82,83]. The mechanisms responsible for the increased IL-8 expression induced by paclitaxel in ovarian cancer cells involve increased expression of toll-like receptors (TLRs) and increased p65 NFκB binding to IL-8 promoter [80,83].

Bortezomib (BZ) is the first FDA approved proteasome inhibitor, which has shown a limited effectiveness in ovarian cancer treatment as a single agent [84,85,86,87]. However, BZ has been considered in combination with cisplatin, since BZ prevents the cisplatin-induced degradation of cisplatin influx transporter, resulting in enhanced cisplatin uptake and tumor cell killing [88,89]. We have recently shown that BZ increases expression of IL-8 and CCL2 in ovarian cancer cells, while it does not affect expression of other NFκB-dependent genes. The responsible mechanisms involve a gene specific and IKKβ-dependent recruitment of S536 phosphorylated p65 NFκB to IL-8 and CCL2 promoters, suggesting that anti-inflammatory therapy targeting IKKβ might increase the BZ effectiveness in ovarian cancer treatment [41]. Since approximately 50% of women diagnosed with ovarian cancer die from chemoresistant metastatic disease, understanding the molecular mechanisms by which chemotherapeutic interventions increase the chemokine expression in ovarian cancer cells should lead to the development of more effective combination strategies.

## 3. Chemokine Transcriptional Regulation in Ovarian Cancer Cells

Chemokines listed in Table 1 have all been identified in ovarian cancer cells and tissues. Various online databases can be used to assess putative transcription factor binding sites. For this review, we have obtained chemokine promoter sequences from the NCBI database and used the Alggen promoter-mapping program to search for the transcription factor binding sites [90,91]. All found putative binding sites are listed in Table 2, Table 3, Table 4 and Table 5; the binding sites that have been experimentally confirmed are highlighted in bold and labeled with an asterisk. Below, we limit discussion of the transcriptional mechanisms only to the chemokines that have been experimentally confirmed in ovarian cancer cells. While the first insights into the chemokine transcriptional regulation were obtained by using *in vitro* electrophoretic mobility shift assays (EMSA) or overexpression experiments, chromatin immunoprecipitations (ChIP) generally provides a more realistic picture about the transcription factor binding to endogenous promoter sequences in living cells.

**Table 2:** List of putative transcription factor binding sites in human CCL2 promoter.

Factor	Site	Sequence	Factor	Site	Sequence
SP-1	-54/-44	ACTCCGCCCT	c-Fos	-1465/-1457	CTGACTCC
Nkx-1	-65/-58	CCTCCTG	p53	-1541/-1534	GGGCAGG
Elk-1	-76/-71	GGAAG	HOX-11	-1571/-1564	CCTAACG
GATA	-88/-82	CTTATC	PEA3	-1644/-1636	AAACATCC
C/EBP	-112/-106	TTGCTC	GR	-1790/-1782	TTGTTCTC
ELF	-143/-130	CTACTTCCTGGAA	AR	-1789/-1781	TGTTCTCT
Hif-1 *	-127/-122	CACAG	FOXP3	-1959/-1950	AAACATTTT
AP-1 *	-139/-131	TTCCTGGAA	C/EBP	-1980/-1973	TTGCACA
STAT1-3 *	-139/-131	TTCCTGGAA	Pbx-1	-2132/-2120	AGCATGACTGGA
C-Ets1	-140/-133	CTTCCTG	FOXO-3	-2184/-2176	CTTATTTA
NF-AT	-181/-172	GGAAAAAGT	CUTL-1	-2309/-2303	ATTGGT
E47	-239/-232	GTCTGGG	PR	-2358/-2351	GAACACT
RP58	-256/-245	GTTCACATCTG	Smad3	-2521/-2511	GAGGCAGACA
HNF-1	-654/-646	TAATATTT	ERα	-2570/-2562	CTGACCTC
TMF	-708/-701	TATAACA	c-Jun	-2580/-2574	CATGGG
HNF-3	-742/-735	CTATTTA	NFκB *	-2600/-2591	GGAATTTCC
AP-2	-747/-741	GCAGGC	ZDX/BCL6	-2632/-2621	GGGAACTTCC
c-Jun	-942/-935	TGACTTA	E47	-2678/-2671	ATCTGGA
HMG1	-1042/-1035	GGAAATT	ETF	-2717/-2708	CACAGCCCC
IRF-3	-1089/-1082	GCTTTCC	GATA	-2902/-2893	CTTTATCT
BTEB3	-1287/-1278	AGGAGGAGG	PU-1	-3041/-3031	TTACTTCCTC
NF-Y	-1315/-1307	ATTGGGCA	YY1	-3264/-3257	AAAATGG
USF-2b	-1447/-1439	GTCATTTG	RAR	-3429/-3421	ATCTCACC

* Experimentally confirmed binding sites, Hif-1; Hypoxia inducible factor-1, AP-1; Activator protein-1, STAT1-3; Signal transducer and activator of transcription 1-3, NFκB; Nuclear factor kappa B.

**Table 3:** List of putative transcription factor binding sites in human CXCL1 promoter.

Factor	Site	Sequence	Factor	Site	Sequence
IRF-3	-50/-43	GCTTTCC	Elk-1	-771/-766	GGAAG
HMG I	-75/-68	AATTTCC	FOXP3	-791/-782	CAACATTTT
MBP-1	-78/-68	GGGAATTTCC	MZF-1	-810/-803	CAGGGGA
NFκB *	-79/-68	CGGGAATTTCC	TGIF	-870/-862	TGACAACC
CDP *	-97/-87	GGGATCGATC	C/EBP	-980/-974	TTGCAC
E47	-90/-83	ATCTGGA	YY-1	-1061/-1054	TAAATGG
E2F-1	-126/-119	GGCGGGG	c-Ets	-1076/-1069	CAGGAAG
SP3	-128/-119	GGGGCGGGG	AR	-1394/-1386	TGTTCTCT
SP-1 *	-130/-121	GGGGGCGGG	c-Jun	-1491/-1483	TGACTCAT
R2	-137/-131	TCCACC	Pax	-1909/-1902	CCTTGAC
LF-A1	-247/-240	TGGGGCA	ERα	-2057/-2050	TGGGTCAA
AP-2 *	-279/-273	GCAGGC	NF-Y	-2060/-2052	ATTGGGTC
AREB6	-296/-288	CAGGTGGT	LEF-1	-2807/-2799	CTTTGTTG
Smad3	-563/-553	TTCACAGACA	HNF-1	-2966/-2958	TAATATTT
PR	-602/-595	GAACATT	RAR	-3102/-3094	ATGCCTTAG
GR	-605/-596	GCAGAACAT	NHP-1	-3103/-3096	TGACCTT
TMF	-739/-732	TGTTATA	PEA3	-3110/-3102	GGATGTAT
GATA	-767/-761	GATAAG	ATF	-3452/-3443	TGACGTAAA

* Experimentally confirmed binding sites, CDP; CAATT displacement protein, SP-1; Specificity protein 1, AP-2; Activator protein 2.

**Table 4:** List of putative transcription factor binding sites in human CXCL2 promoter.

Factor	Site	Sequence	Factor	Site	Sequence
NFκB *	-76/-67	GGGAATTTCC	BTEB3	-862/-853	AAGCGGAGT
CREB	-83/-74	CGGACGTCA	NF-Y	-970/-962	GAACCAAT
ATF-2	-83/-74	CGGACGTCA	HMG I	-999/-992	AATTTCC
HLF	-104/-95	GTTACGCAA	IRF	-999/-992	AATTTCC
E2F-1	-111/-104	GGCGGGA	NF-AT	-1001/-992	AAAATTTCC
NF-1	-113/-108	TTGGC	CUTL1	-1085/-1079	ATTGAT
LF-A1	-139/-132	CGGGGCA	FOXP3	-1115/-1106	CTTAATTTT
GATA	-192/-184	GGTTATCT	PR A	-1257/-1250	GAACACT
AP2α	-198/-192	GCAGGC	C/EBP	-1367/-1360	TGAGCAA
STAT3 *	-218/-210	TTGGGGAA	MZF1	-1380/-1373	CAGGGGA
ERα	-241/-233	CTGACCCA	HNF-1	-1440/-1432	ATATTAAC
PEA3	-276/-268	GGATGTAG	TMF	-1880/-1873	TATAACA
Elk-1	-296/-292	GAAG	E47	-1830/-1823	TTCTGGA
STAT3 *	-318/-310	GGGATCGATC	Nkx2	-1827/-1820	CTGGAGG
p53	-339/-332	CTTGCCC	HNF	-2153/-2146	TAAATGG
AhR	-418/-410	GCGTGCGT	YY1	-2153/-2146	TAAATGG
c-Jun *	-437/-430	TGACACA	HSF1	-2409/-2401	ATTCTAGG
c-Fos	-451/-443	TGCGTCAT	ETF	-2505/-2496	GGGGCTGTC
c-Ets	-473/-467	CAGGAAG	AP3	-2636/-2629	GAGTTAG
USF-1	-508/-499	ACACGTGAT	Smad3	-3112/-3102	CAGTCAGACA
AREB6	-574/-566	AACACCTG	LEF-1	-3101/-3093	CAACAAAG
FOXJ2	-621/-611	AAAATAAACA	TCF-1	-3102/-3093	ACAACAAAG
AR	-673/-665	TGTTCCAA	GR	-3256/-3247	ACAGAACAT

* Experimentally confirmed binding sites, c-Jun; Jun proto-oncogene.

**Table 5:** List of putative transcription factor binding sites in human CXCL8 promoter.

Factor	Site	Sequence	Factor	Site	Sequence
NFκB *	-80/-70	GGAATTTCC	E47	-859/-852	ATCTGGA
PU-1	-83/-73	GGAATTTCCTC	PR	-868/-861	ACTCTTC
NRF *	-88/-77	ATTCCTCTGA	HSF1	-867/868	CCTTGAAT
C/EBP *	-94/-87	TTGCAAA	IRF	-973/-964	TTTCCATTA
MZF-1	-112/-105	GAGGGA	RAR	-1068/-1061	AGAGGTC
EBF	-118/-107	TGCCCTGAGGG	ERα	-1067/-1060	GAGGTCA
C/EBP *	-119/-112	TTGCACA	p53	-1258/-1251	CTTGCCC
AP-1 *	-129/-121	TGACTCAG	FOXP3	-1304/-1295	AAAATGAAG
c-Ets	-141/-132	TAGGAAGTC	RelA	-1367/-1357	GGCATTCCCC
Elk-1	-139/-134	GGAAG	YY1	-1372/-1365	AAAATGG
LEF-1	-187/-179	GATCAAAG	Smad3	-1403/-1393	GAAACAGACA
Hif-1 *	-234/-229	GTGCG	Nkx1	-1457/-1450	CCTCAAG
GRα	-335/-327	TTGTTCTA	AP2α	-1473/-1467	CCAGGC
AREB6	-328/-320	AACACCTG	TCF1	-1663/-1654	ACAACAAAG
AR	-334/-326	TGTTCTAA	NF-AT	-1687/-1677	CTAATTTTCC
NF	-424/-416	ATTGGCTC	HMGI	-1685/-1677	AATTTTCC
AP3	-535/-528	TAAATC	HLF	-1695/-1686	TTGTGTAAC
HNF-3	-606/-599	TAAATGT	CUTL1	-1858/1852	TTGGT
FOXO3	-651/-641	CTTATCTA	PEA3	-2174/-2166	GCACATCC
GATA	-651/-644	CTTTATCT	HOX11	-2200/-2193	CGTTAGG
c-Myb	-792/-784	CAACTGCC	RARγ	-2225/-2217	GGCTCACC
C/EBP	-798/-792	TTGCTC	AIRE	-2555/-2545	ATGGTTATCT
GR	-847/-838	CTGTTCTCT	Oct1	-2744/-2733	TCACTTTGCAT

* Experimentally confirmed binding sites, C/EBP; CCAAT enhancer binding protein, NRF; NFκB repressing factor.

### 3.1. CCL2

**Figure 1:**
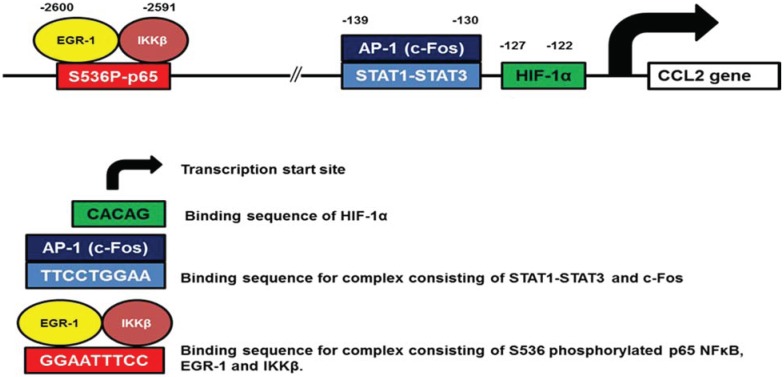
Schematic illustration of human CCL2 promoter.

CCL2 (MCP-1) is an important determinant of macrophage infiltration in ovarian tumors [92,93]. Although CCL2 has been originally thought to have an inhibitory effect on ovarian cancer progression [94,95,96], recent studies have indicated that CCL2 increases invasion of ovarian cancer cells and resistance to chemotherapy [97,98]. The putative transcription factor binding sites identified in human CCL2 promoter are listed in Table 2. Experimental studies demonstrated binding of NFκB, STAT1, STAT3, AP-1, and Hif-1α to the CCL2 promoter in OC cells (Figure 1).

Even though the NFκB binding site is located in the distal regulatory region of human CCL2 promoter (Figure 1), several studies have demonstrated p65 NFκB involvement in the regulation of CCL2 expression in OC cells [27,41,99]. In addition, CCL2 expression is regulated by IKKβ-dependent recruitment of the transcription factor EGR-1, and inhibition of IKKβ activity decreases p65 and EGR-1 promoter recruitment and CCL2 expression [41]. Interestingly, the NFκB binding site in human CCL2 promoter has the same nucleotide sequence as the NFκB site in human IL-8/CXCL8 promoter. Curiously, both CCL2 and IL-8 are increased by paclitaxel [83] and bortezomib [41], indicating that the paclitaxel and BZ-induced CCL2 (and IL-8) increase is promoter specific.

Activity of the transcription factors STAT-1 and STAT-3 is also constitutively increased in OC cells, where it promotes cell motility and invasiveness [100]. Phosphorylation of STAT3 at tyrosine residues 705 and 727 increases its transcriptional activity [101]. In OC cells, IL-6 [102] and M-CSF [103] induce phosphorylation and activation of STAT3, and increase the CCL2 expression. In addition to NFκB and STAT transcription factors, studies in other cell types indicated that the CCL2 expression is positively regulated by AP-1 and Hif-1α [104,105,106,107].

Though no transcription factors have been reported to be involved in the negative regulation of CCL2 in OC cells, studies involving other cell types have reported negative regulators of CCL2. Specifically, NFκB p50/p50 homodimers, HDAC1, and the transcription factors Nrf2 and SMRT have been suggested to suppress the CCL2 expression in hepatic cells and adipocytes [108,109,110].

### 3.2. CXCL1

**Figure 2:**
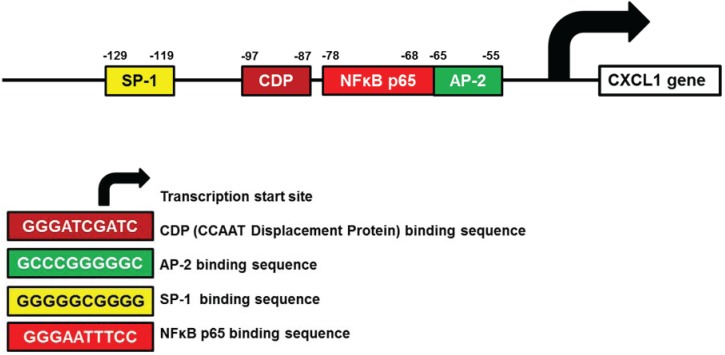
Schematic illustration of human CXCL1 promoter.

CXCL1 (GRO-α) contributes to ovarian cancer progression by inducing endothelial and epithelial cell proliferation and migration [25,26]. The putative transcription factor binding sites identified in human CXCL1 promoter are listed in Table 3. Experimental studies have demonstrated binding of the transcription factors p65 NFκB, AP-2, CCAAT displacement protein (CDP), and the stimulating protein-1 (SP-1) to the CXCL1 promoter in human cells (Figure 2). In ovarian cancer cells, though, the CXCL1 gene expression was found to be regulated mainly by NFκB pathway, specifically by the p65 DNA binding [25,27,28,111,112].

In addition to the positive regulation by p65 NFκB, AP-2 and SP-1, studies using human melanocytes have indicated that the CXCL1 expression is negatively controlled by the transcriptional repressors CDP and the poly(ADPribose) polymerase-1 (PARP-1) [113,114]. The exact mechanisms of how CDP and PARP-1 inhibit the CXCL1 expression are not fully understood; however, they likely involve displacement of trans-activating factors that bind to CXCL1 promoter, resulting in transcriptional repression.

### 3.3. CXCL2

**Figure 3:**
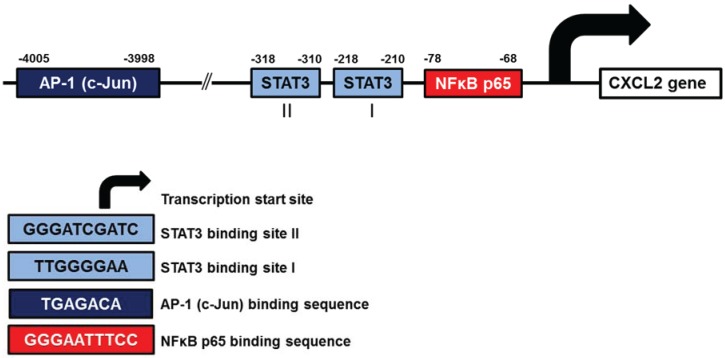
Schematic illustration of human CXCL2 promoter.

The putative transcription factor binding sites identified in human CXCL2 (GRO-β) promoter are listed in Table 4. However, experimental studies have demonstrated only binding of NFκB, AP-1, and STAT3 to human CXCL2 promoter (Figure 3). In ovarian cancer cells, the CXCL2 expression is dependent on IκBα [28] and IKKβ [44]. In addition, the CXCL2 expression in OC cells is induced by TNF, and is inhibited by overexpression of the tumor suppressor p53 [115].

### 3.4. CXCL8

**Figure 4:**
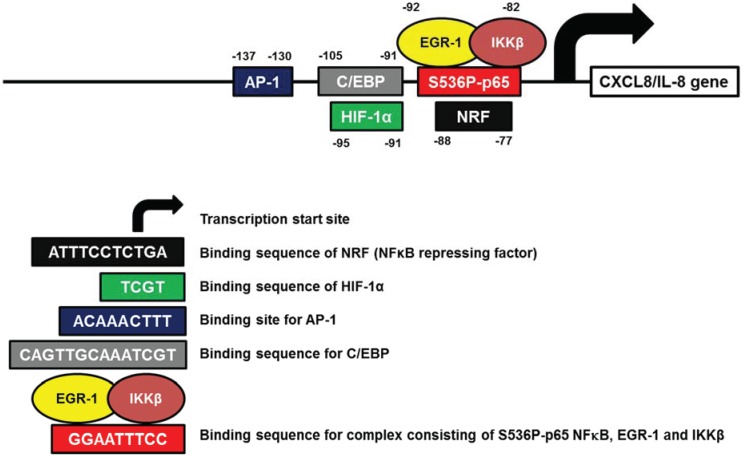
Human CXCL8 promoter with the identified transcription factor binding sites.

CXCL8 (IL-8), an inflammatory chemokine originally discovered as the neutrophil chemoattractant and inducer of leukocyte-mediated inflammation [1,2,3], contributes to cancer progression through its induction of tumor cell proliferation, migration and angiogenesis [4,5,6,7,8,9]. The expression levels of IL-8 directly correlate with ovarian cancer progression, and suppression of IL-8 expression inhibits angiogenesis and tumorigenicity of ovarian cancer cells [13,116,117,118]. A number of studies have identified a minimal region in human IL-8 promoter that spans nucleotides -1 to -140, is necessary for IL-8 transcription, and contains binding sites for NFκB, AP-1, CCAAT enhancer-binding protein beta (C/EBP or NF-IL6), Hif-1, and NFκB-repressing factor (NRF) [119,120,121,122,123,124,125,126,127]. In addition, the IL-8 transcription in ovarian cancer cells is positively regulated by the transcription factor early growth response-1 (EGR-1) binding to IL-8 promoter, and by enzymes of IKK complex that phosphorylate both IκBα, leading to its cytoplasmic degradation, and p65 NFκB, resulting in its increased transcriptional activity (Figure 4) [41,42,43,44,45].

NFκB is crucial for the IL-8 expression, and regulates IL-8 in all cell types [128]. The NFκB binding sequence (GGAATTTCC) is located between -80 and -70 of the IL-8 gene [120]. In most cell types, the IL-8 transcription is regulated predominantly by p65 homodimers [37,121,129,130,131]. Phosphorylation of p65 NFκB on serines 276 and 536 increases its transcriptional activity and interaction with other transcription factor and regulators, and decreases its affinity for nuclear IκBα [129,130,131,132,133]. We have recently shown that in ovarian cancer cells, the IL-8 transcription is regulated by S536-p65 NFκB, IKKβ, and EGR-1, and that proteasome inhibition developed as a strategy to inhibit NFκB-dependent transcription, paradoxically increases the IL-8 expression in ovarian cancer cells by increasing the S536-p65, IKKβ and EGR-1 recruitment to IL-8 promoter [41].

Adjacent to the NFκB site in the IL-8 promoter are C/EBP and Hif-1 binding sites (Figure 4). Even though the direct involvement of C/EBP and Hif-1 in the IL-8 regulation in ovarian cancer cells has yet to be demonstrated, the up-regulation of IL-8 expression by hypoxia in ovarian cancer cells has been well documented [30,134].

Transcription of IL-8 is also regulated by the transcription factor AP-1 that consists of Fos, FosB, Jun, and Jun-B subunits. Activation of AP-1 mediates the increased IL-8 expression in hypoxia, paclitaxel, and lysophosphatidic acid (LPA) treated OC cells [30,80,135]. Interestingly, a recent study has shown that the stress hormones norepinephrine and epinephrine enhance the IL-8 expression by a FosB-dependent mechanism [136]. Table 5 lists all putative transcription factor binding sites identified in the human CXCL8/IL-8 promoter.

Although studies from other cell types have shown that the IL-8 expression is negatively regulated by the NFκB repressing factor NRF, nuclear receptor corepressor (NCoR), the silencing mediator for retinoic acid and thyroid hormone receptor SMRT, and HDACs [54,137,138,139], the potential involvement of these corepressors in OC cells has yet to be demonstrated. Considering the important role these corepressors play in the IL-8 regulation, it will be important to elucidate their function in ovarian cancer setting.

## 4. Conclusions and Perspectives

As we continue to improve our understanding of the mechanisms regulating chemokine expression in ovarian cancer cells, our knowledge will contribute to the development of new therapeutic strategies targeting the increased chemokine expression in chemoresistant metastatic ovarian cancer. Several important questions remain to be answered: What are the specific molecular targets and mechanisms responsible for the chemokine expression induced by chemotherapeutic drugs and hypoxia? What is the role of HDACs and other transcriptional repressors in regulating the chemokine expression in ovarian cancer cells? What is the role of the metabolic state of ovarian cancer cells in regulating the chemokine expression? Answers to these questions may open new avenues for therapeutic approaches for treating ovarian cancer.
